# A case of gastric metastasis from renal cell cancer during the sequential targeted therapy

**DOI:** 10.1007/s13691-017-0286-x

**Published:** 2017-04-04

**Authors:** Sho Uehara, Takeshi Yuasa, Junko Fujisaki, Yasuhisa Fujii, Shinya Yamamoto, Hitoshi Masuda, Iwao Fukui, Junji Yonese

**Affiliations:** Department of Urology, Cancer Institute Hospital, Japanese Foundation for Cancer Research, 3-8-31 Ariake, Koto-ku, Tokyo, 135-8550 Japan

**Keywords:** Renal cell carcinoma, Gastric metastasis, Targeted therapy

## Abstract

Historically, gastric metastasis from renal cell cancer (RCC) has been extremely rare. As RCC is now being treated with various agents of targeted therapy, however, the rate of unusual visceral metastases might increase as outcomes improve and follow-up periods grow longer. Here we present a valuable case of gastric metastasis from RCC detected in a routine CT scan during sequential targeted therapy. A 73-year-old man was referred to our hospital, presenting with left renal cell cancer with multiple lung metastases at stage cT1bN0M1. He received first-line targeted therapy consisting of sunitinib and subsequently underwent left radical nephrectomy. He then underwent sequential therapy consisting of interferon-alpha, sunitinib, everolimus, and axitinib for multiple lung metastases. Five years after nephrectomy, a follow-up computed tomography scan revealed a 2.2 × 1.6 cm mass in the stomach without any symptoms. Gastrointestinal endoscopy disclosed a polypoid lesion at the gastric fundus. Endoscopic submucosal resection was performed. Microscopic diagnosis revealed gastric metastasis from RCC. As various new therapeutic agents increase survival periods for metastatic RCC patients in this era of targeted therapy, clinicians must watch for metastasis in the stomach, though this was formerly a rare event.

## Introduction

In patients with metastatic renal cell cancer (RCC), lung, bone, and liver are common metastatic sites, with frequency estimates around 50, 30, and 20%, respectively [1, 2]. Gastric metastases from RCC are rare, with a frequency between 0.2 and 0.7% reported in clinical and autopsy series [3, 4]. In clinical practice, however, RCC is one of the most frequent primary cancer types, third after breast and lung cancers, from which metastatic gastric tumors originate [5, 6]. In recent years, various new targeted agents have become available for the treatment of metastatic RCC. Not only the survival period but also the duration of treatment, which includes suitable scheduled imaging procedures, have been extended. Consequently, the rate at which formerly unusual visceral metastases are seen during follow-up might increase over the next few years. Here, we report a case of gastric metastasis from RCC detected in a routine computed tomography (CT) scan during sequential targeted therapy.

## Case report

**Fig. 1:**
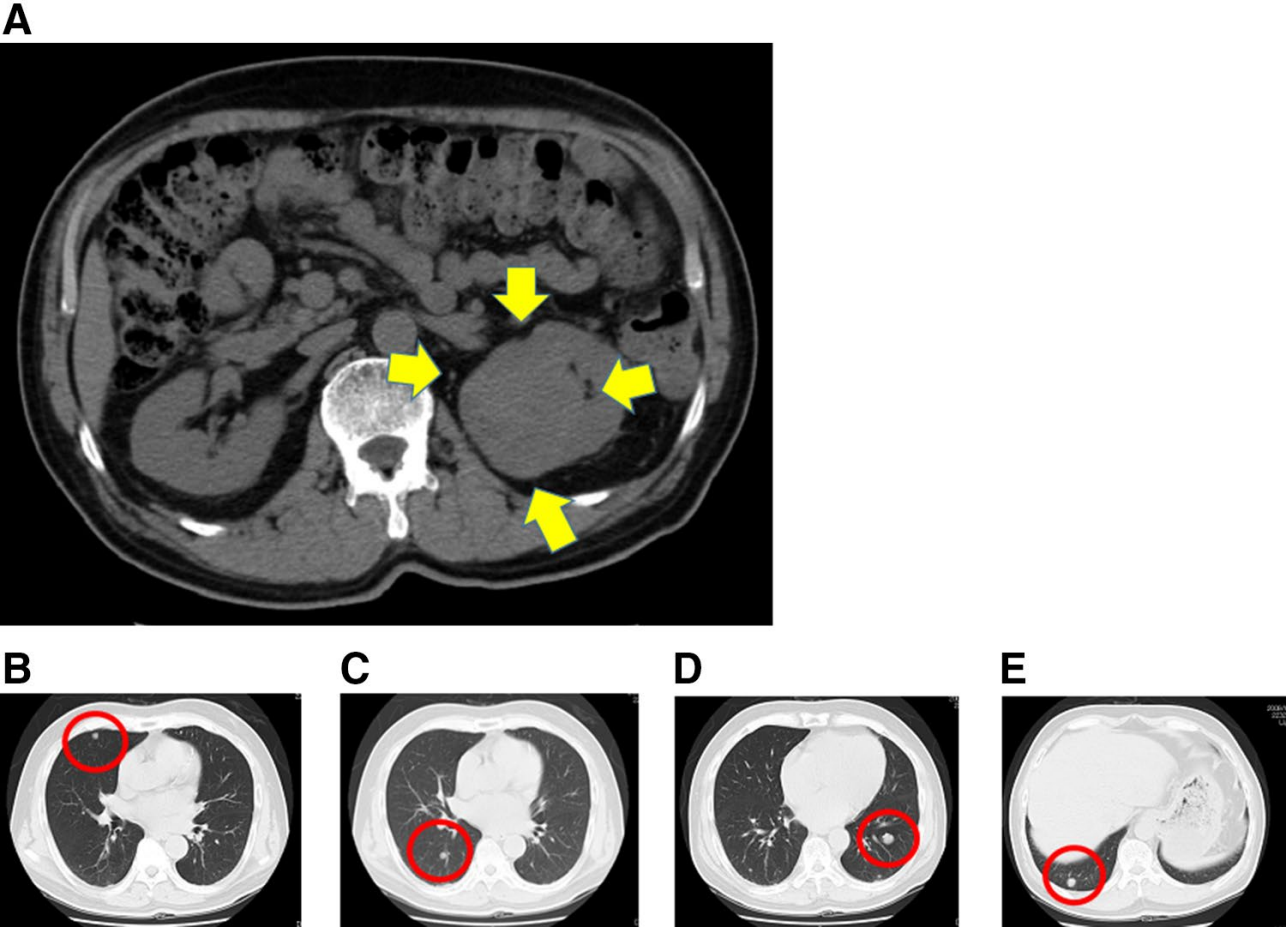
Initial presentation of a case of metastatic renal cell cancer. Primary tumor in the *left* kidney (**a**). Multiple lung metastatic lesions (**b**–**e**)

**Fig. 2:**
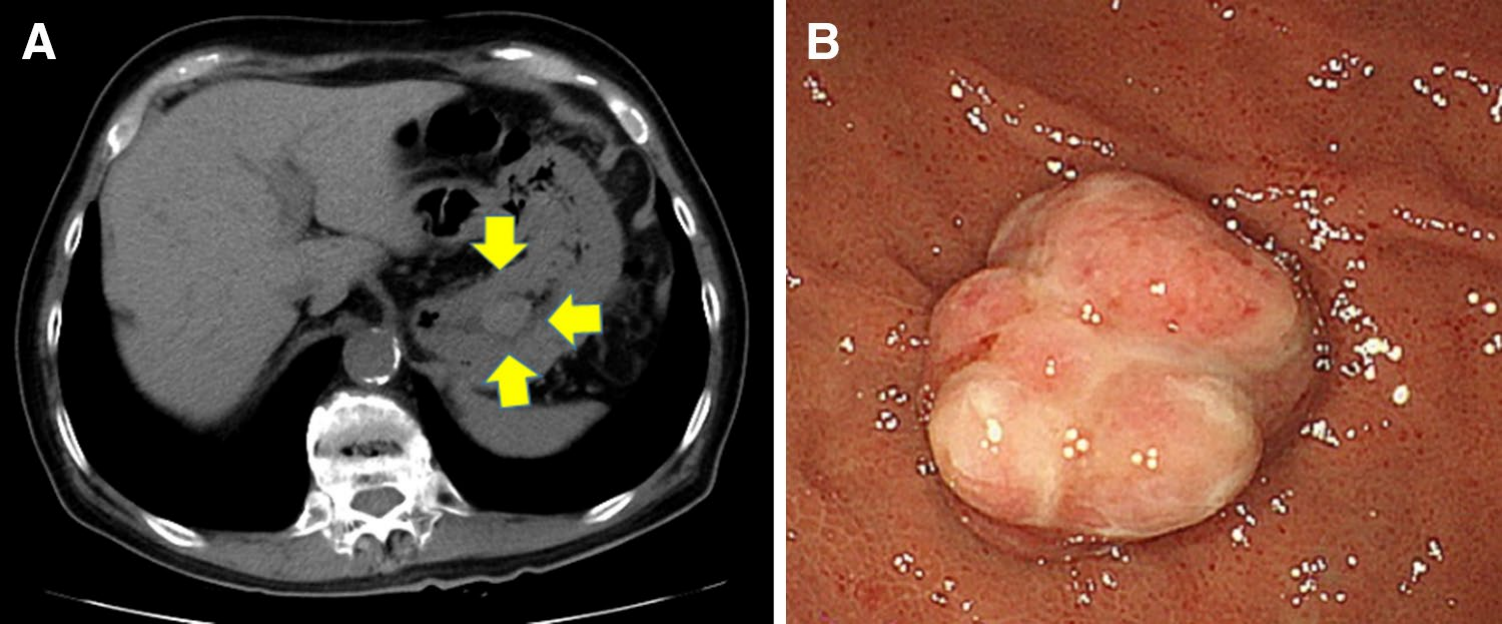
Gastric metastasis from renal cell cancer. Routine follow-up CT scan revealed a tumor lesion in the stomach (**a**). Endoscopic appearance of a solitary, distinct polypoid lesion (**b**)

In June 2009, a 73-year-old man was referred to our hospital presenting with left RCC measuring 6.0 × 5.6 cm with multiple lung metastases (Fig. 1a–f). The patient had a history of diabetes mellitus and allergy to iodine-containing contrast medium. At his previous clinic, he had been given a transbronchial lung biopsy and diagnosed with clear cell RCC metastatic to the lung. At our hospital, thoracoabdominal CT scan and bone scintigraphy showed no other visceral or lymph-node metastases, and the patient was diagnosed with clear cell RCC with cT1bN0M1 staging. He received sunitinib as the first-line systemic therapy for metastatic clear cell RCC. Eight months later, the primary renal tumor was reduced to 5.5 × 4.8 cm. In addition, the volume of the metastatic sites was also reduced (the maximum nodule decreased from 1.5 × 1.3 to 1.0 × 0.8 cm). Because we could confirm the responses of medical therapy and the patient had a good performance status, cytoreductive nephrectomy was performed in May 2010. The pathological investigation revealed well-defined (G1-2) clear cell RCC measuring 4.5 × 4.0 × 2.0 cm in the upper pole of the left kidney, which had a negative margin and negative lymphovascular invasion. Eosinophilic amorphous materials, which was a degenerative effect of the previous therapy, were found in the tumor. After nephrectomy, the patient received interferon-alpha cytokine therapy for 13 months because his metastatic sites were limited to lung. However, his lung metastases continued to grow gradually during this treatment, targeted therapy with sunitinib was re-introduced. Between June 2011 and January 2016, he underwent sequential targeted therapy consisting of sunitinib, everolimus, and axitinib for 21, 11, and 23 months, respectively. In October 2015, a follow-up CT scan disclosed a mass measuring 2.2 × 1.6 cm in the stomach (Fig. 2a). The patient had experienced no symptoms associated with the gastric mass. Upper gastrointestinal endoscopy revealed a solitary, distinct polypoid lesion (2 cm in diameter) classifiable as a 0-I tumor according to the Japanese Classification of Gastric Carcinoma (14th edition) [7] at the fundus of the stomach (Fig. 2b). Tumor biopsy was performed and microscopic examination confirmed that this lesion was a metastasis from RCC. In February 2016, the patient underwent endoscopic mucosal resection of the gastric metastatic lesion. Histological evaluation of the resected specimen (3 cm in diameter) demonstrated well-defined clear cell RCC histology (G1 > G2) with submucosal invasive depth, positive vertical margin, and negative lymphovascular invasion. The patient’s therapy was switched to immunotherapy with the anti-programmed cell death 1 antibody nivolumab (2 mg/kg every 3 weeks). Six months after initiation of the immunotherapy, follow-up CT scan showed that the lung metastases had diminished remarkably and no new additional metastasis was found. Regarding the gastric lesion, follow-up endoscopy and endoscopic ultrasonography were performed 1 month after resection. There were no residual tumors although there were positive margins. Thereafter, these endoscopic examinations were performed every 6 months. To date, there has been no recurrence despite the positive surgical margin.

## Discussion

Gastric metastasis is a rare finding. Its reported primary tumor types are breast cancer (27%), lung cancer (23%), RCC (7.6%), and malignant melanoma (7%) [5, 6]. Among the various possible metastatic sites from RCC, the stomach has only rarely been reported [8–14]. Cabezas-Camerero et al. reviewed 62 cases of gastric metastases from renal cell carcinoma [8]. Median age at diagnosis of gastric metastasis was 66.5 years (range 38–87 years) with a male-to-female ratio of 45:17. Bleeding, melena, hematemesis, and abdominal pain were reported in 56.5, 82.9, 25.7, and 19.7% of patients, respectively. There were single lesions in 71.4% and multiple (≥2) lesions in 28.6% of patients. Median size was 30 mm (range 5–100 mm). At the time of gastric metastasis, 66% had metastases in other organs, mainly in lung, bone, lymph nodes, pancreas, and brain. Median interval from diagnosis of RCC to gastric metastasis was 4.5 years (range 0–24 years). Treatment for gastric metastasis was reported in 56 patients and consisted of a surgical procedure in 44.6%, an endoscopic procedure in 28.6%, and others (systemic therapy, radiotherapy, vascular embolization, no treatment) in 27%. Cabezas-Camerero et al. showed a clear tendency toward less aggressive therapies for endoscopic resection after 2004. The interval from diagnosis of gastric metastasis until death was only reported in 25 cases, and it was generally short, with a median of 4 months (range 1–72 months). In our case, asymptomatic gastric metastasis was found 6.6 years after RCC diagnosis during a routine follow-up CT scan. To our knowledge, this is the first case in which gastric metastasis was detected by CT scan. Because of the various effective agents, the survival period of patients with metastatic RCC has been extended. We assume that formerly rare metastasis sites could increase. Therefore, systematic evaluation by routine follow-up CT scan could become more important. Our patient is of an advanced age but with good performance status; although he has multiple lung metastases, they are well managed using various targeted agents. Due to the development of effective medical agents, we chose endoscopic excision with very low invasiveness. After endoscopic excision of the gastric metastasis, the patient started nivolumab therapy. This may be the first report of nivolumab therapy for gastric metastasis as well. After 6 months of treatment and observation, thoracoabdominal CT scan disclosed that the lung metastasis had shrunk remarkably and gastric endoscopy found no progression.

In conclusion, we reported a RCC gastric metastasis that was detected in a routine follow-up CT scan. Because the development of various new effective targeted agents extends the survival period for metastatic RCC patients, events that were formerly considered to be rare could occur.
